# Efficacy and safety of supraglottic jet oxygenation and ventilation in Chinese patients undergoing bronchoscopic procedures: a systematic review and meta-analysis

**DOI:** 10.3389/fmed.2026.1721209

**Published:** 2026-02-24

**Authors:** Mingyuan Yang, Hong Li, Qinghao Cheng

**Affiliations:** Department of Anesthesiology, Emergency General Hospital, Beijing, China

**Keywords:** bronchoscopy, hypoxemia, meta-analysis, supraglottic jet oxygenation and ventilation, systematic review

## Abstract

**Purpose:**

Hypoxemia remains a major complication during bronchoscopic procedures. This systematic review and meta-analysis is to systematically evaluate the efficacy and safety of supraglottic jet oxygenation and ventilation (SJOV) in preventing hypoxemia and improving clinical outcomes during bronchoscopy procedures.

**Methods:**

Databases including Embase, MEDLINE, PubMed, Cochrane Library, Weipu, SinoMed, Wanfang Data and China National Knowledge Infrastructure were searched for randomized studies comparing SJOV with other conventional oxygen therapy in adult patients undergoing diagnostic or therapeutic bronchoscopy. The primary outcome was the incidence of hypoxic events. Secondary outcomes included the need for airway assistance, arteria blood gas, peri-operative adverse events.

**Results:**

A total of 1,090 records were screened, and 10 RCTs involving 1,109 patients (555 in the SJOV group, 554 in the control group) were included, all conducted in China. SJOV significantly reduced the incidence of hypoxemia (OR = 0.20, 95%CI [0.07–0.58], *I*^2^ = 80%), improved mean intraoperative oxygen saturation (SpO₂) (2.27, [0.12–4.42], 99%) and arterial oxygen partial pressure (PaO₂) compared with control group (36.31, [6.16–66.46], 99%). SJOV substantially reduced the need for jaw lift (0.09, [0.03–0.30], 79%), adjustment of ventilator parameters (0.05, [0.03–0.12], 45%), and mask ventilation with positive pressure (0.04, [0.01–0.12], 0%). SJOV was associated with a higher incidence of post-procedural xerostomia (6.08, [2.99–12.36], 44%) and sore throat (1.71, [1.08–2.71], 0.00), but no significant difference in nasal bleeding (0.70, [0.39–1.25], 0%). Intraoperatively, SJOV showed lower tachycardia (0.35, [0.14–0.91],15%) and reduced hypertension incidence (OR = 0.20, [0.06–0.70], 45%).

**Conclusion:**

SJOV significantly improves oxygenation, reduces hypoxemia and rescue interventions during bronchoscopy, with a manageable safety profile. It may be a valuable oxygenation strategy for bronchoscopic procedures, especially in high-risk patients.

**Systematic review registration:**

https://www.crd.york.ac.uk/PROSPERO/view/CRD420251083408.

## 1 Introduction

Bronchoscopic procedures have become indispensable in the diagnosis and treatment of respiratory diseases, enabling interventions ranging from biopsy and bronchoalveolar lavage to tumor ablation and stent placement (1–4). Despite their utility, procedural hypoxemia remains the most frequent and hazardous complication, with reported incidence ranging from 30% to as high as 70% (5–12). This complication arises from multifactorial mechanisms, including sedative-induced respiratory depression, airway obstruction caused by the bronchoscope itself, airway irritation leading to laryngospasm or bronchospasm, and ventilation-perfusion mismatch caused by procedural manipulations (13–16). Such severe hypoxemia may force premature termination of the procedure and can precipitate arrhythmias, cardiac arrest, or irreversible end-organ injury, contributing to up to 25% of anesthesia-related deaths (17).

Current commonly used oxygenation strategies, such as nasal cannula, face mask, high-flow nasal cannula (HFNC), and even non-invasive ventilation (NIV) or mechanical ventilation via a laryngeal mask airway, often fail to meet the increased oxygen demand during bronchoscopy, particularly in high-risk patient cohorts, such as those with obesity, advanced chronic obstructive pulmonary disease (COPD), elderly individuals, or during prolonged procedures (18–22). These techniques are limited by their inability to effectively flush the anatomical dead space or to generate sustained positive end-expiratory pressure (PEEP), resulting in persistent alveolar hypoventilation and recurrent desaturation. While advanced oxygenation techniques like HFNC and NIV, have shown promise in reducing hypoxemia risk, their application in bronchoscopy is constrained by patient tolerance, equipment complexity, and interference with procedural access (17, 21–23).

In this context, supraglottic jet oxygenation and ventilation (SJOV) has emerged as a promising alternative, demonstrating significant clinical benefits in reducing the incidence of hypoxemia and consistently improving oxygenation parameters across studies (24–27). This technique delivers high-frequency, high-pressure oxygen pulses via a catheter positioned above the glottis, which effectively flushes pharyngeal dead space and generates functional PEEP without obstructing the endoscopic field, thus theoretically addressing the key limitations of conventional oxygen therapy (24–26, 28). However, existing studies on SJOV in bronchoscopy have yielded inconsistent results, with variations in patient populations, procedural types, and outcome definitions. A systematic synthesis of evidence is therefore crucial to clarify its efficacy and safety.

To address this evidence gap, we conducted a systematic review and meta-analysis of all available randomized controlled trials (RCTs) to quantitatively synthesize the existing evidence and provide robust conclusions regarding the effectiveness of SJOV for preventing hypoxemia and improving clinical outcomes during bronchoscopic procedures in adults.

## 2 Methods

### 2.1 Study registration

This systematic review and meta-analysis was prospectively registered in the International Prospective Register of Systematic Reviews (PROSPERO) under the registration number CRD420251083408 on June 29, 2025, adhering to the Preferred Reporting Items for Systematic Reviews and Meta-Analyses (PRISMA) guidelines (29).

### 2.2 Search strategy and data sources

A comprehensive literature search was performed to identify all RCTs comparing SJOV with conventional oxygen therapy in sedated patients undergoing procedural interventions. The following databases were searched from their inception to June 2025: PubMed, MEDLINE, EMBASE, Cochrane Central Register of Controlled Trials (CENTRAL), Weipu Data, Chinese Biomedical Literature Service System (SinoMed), Wanfang Data and China National Knowledge Infrastructure (CNKI). The search strategy combined controlled vocabulary and free-text terms related to “supraglottic jet oxygenation and ventilation”, “bronchoscope”, and “randomized controlled trial”. No language restrictions were applied. Reference lists of eligible studies and relevant systematic reviews were manually screened to identify additional studies.

### 2.3 Inclusion and exclusion criteria

#### 2.3.1 Inclusion criteria

Adult patients (≥18 years) undergoing procedural sedation (diagnostic or therapeutic); Intervention group receiving SJOV; Control group receiving conventional oxygen therapy (including nasal cannula oxygen inhalation, mask ventilation); At least one ventilation/oxygenation-related outcome measure (including SpO₂ minimum, incidence of hypoxic events, PaO₂/PaCO₂ change) was reported; RCTs reporting relevant outcomes with full-text availability.

#### 2.3.2 Exclusion criteria

Animal studies, simulation studies, or non-randomized designs; As well as reviews, conference abstracts, case reports, and duplicate publications; Subjects were non-adults (<18 years old) or did not receive bronchoscopy; Patients under general anesthesia with tracheal intubation; Non-SJOV was the intervention method, or the oxygenation method of the intervention and the control group could not be clearly distinguished; The control group was also the SJOV.

### 2.4 Study selection and data extraction

Two reviewers independently screened titles and abstracts to identify potentially eligible studies. Full texts of these studies were then assessed for eligibility using the predefined criteria. The extracted contents include: Name of the first author, year of publication, country/region, type of study design, sample size, age and sex of the subjects, type of bronchoscopy (diagnostic or therapeutic), type of anesthesia, oxygenation/ventilation strategies of the intervention and control groups, primary and secondary outcome indicators and their specific data formats.

For oxygenation-related outcomes, including intraoperative SpO₂ and PaO₂, data were extracted at predefined intraoperative time points, as reported in the original studies. All included studies reported oxygenation parameters using fixed intraoperative measurements rather than minimum values. Differences in measurement timing across studies were recorded and considered as a potential source of heterogeneity.

In case of incomplete data or graphic data, try to contact the author or use image data extraction tools such as Engauge Digitizer. Disagreements were resolved by consensus or consultation with a third reviewer.

### 2.5 Risk of bias assessment

The methodological quality of included RCTs was evaluated using the revised Cochrane Risk of Bias Tool (RoB 2). Two reviewers independently assessed five domains: Bias arising from the randomization process; Bias due to deviations from intended interventions; Bias due to missing outcome data; Bias in measurement of outcomes; Bias in selection of reported results. Each domain was categorized as “low risk,” “some concerns,” or “high risk,” with an overall risk of bias judgment for each study. Disagreements were resolved by consensus.

### 2.6 Certainty of evidence

The Grading of Recommendations Assessment, Development, and Evaluation (GRADE) approach was used to rate the certainty of evidence for each outcome as high, moderate, low, or very low. Factors considered included risk of bias, inconsistency (*I*^2^ statistic), indirectness, imprecision, and publication bias.

### 2.7 Statistical analysis

Data were analyzed using Review Manager 5.4 and Comprehensive Meta-Analysis 4.

Dichotomous outcomes were expressed as odds ratios (OR) with 95% confidence intervals (CI). Continuous outcomes were summarized as mean differences (MD) with 95% CI. A random-effects model was used for all analyses due to anticipated clinical and methodological heterogeneity. Heterogeneity was quantified using the *I*^2^ statistic (*I*^2^ > 75% indicated substantial heterogeneity). Sensitivity analyses (leave-one-out method) were performed to assess result robustness. A two-sided *p* < 0.05 was considered statistically significant.

Subgroup analyses were prespecified and conducted only for the primary outcome to explore potential sources of heterogeneity. Subgroup analyses were not performed for secondary outcomes because of the limited number of available studies.

Subgroup analyses based on the control group were conducted according to the oxygen delivery or ventilation strategies employed in the comparator arms. Control interventions were categorized as: (1) Conventional oxygen delivery, including nasal cannula and endoscopic face mask; and (2) Advanced airway or ventilation strategies, including laryngeal mask airway ventilation, Wei Nasal Jet^®^ (WNJ), oral or nasopharyngeal airway devices.

Subgroup analyses based on the SJOV parameter were conducted according to driving pressure and driving frequency. For driving pressure, reported values were standardized to kilopascals (kPa) when feasible. Owing to the lack of established consensus thresholds for SJOV driving pressure, a data-driven median split approach was applied to classify studies into low-pressure (≤120 kPa) and high-pressure (>120 kPa) groups. Studies with missing or implausible parameters values as reported in the original publications were classified as pressure not reported (NR). Sensitivity analyses were performed by excluding studies with unreported SJOV parameters to assess the robustness of subgroup findings.

## 3 Results

### 3.1 Study characteristics and selection

**Figure 1:**
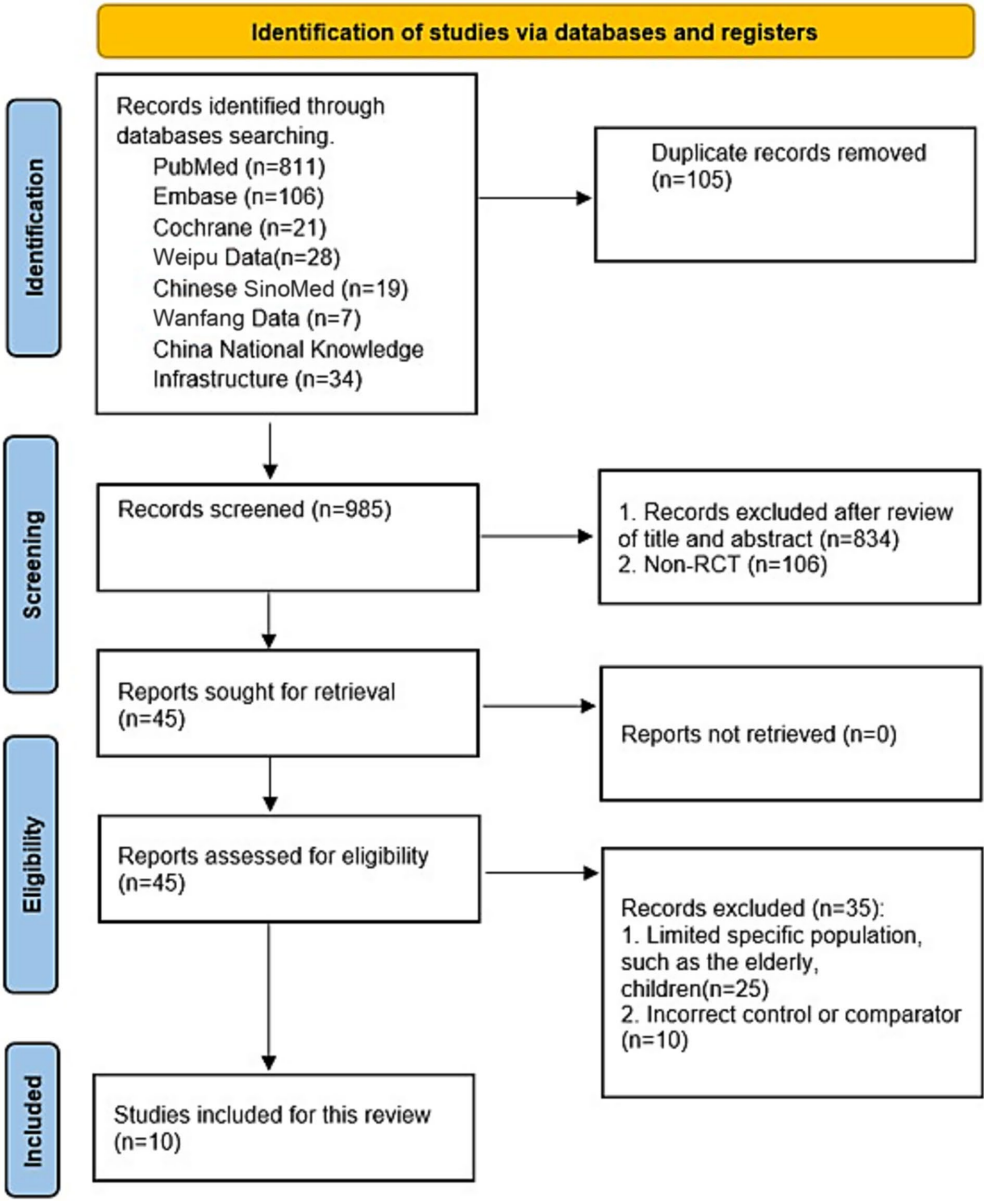
Flow chart of study selection.

Totaling 1,090 initial records were searched from PubMed (*n* = 811), Embase (*n* = 106), Cochrane Library (*n* = 21), Weipu Data (*n* = 28), Chinese SinoMed (*n* = 19), Wanfang Data (*n* = 71) and CNKI (*n* = 34). After removing 105 duplicate entries, 985 records underwent title and abstract screening. Through this systematic process, 940 records that failed to meet basic inclusion thresholds were initially filtered out, advancing 45 reports for full-text retrieval and eligibility assessment. Of these 45 reports, 35 were excluded for the following reasons: restriction to specific populations such as the elderly or children (*n* = 25), and incorrect control or comparator usage (*n* = 10). Ultimately, 10 studies were included in this meta-analysis, as visualized in the study selection flowchart (Figure 1).

A total of 10 randomized controlled trials (RCTs) involving 1,109 patients were included, with 555 patients in the supraglottic jet oxygenation and ventilation (SJOV) group and 554 in the control group. Baseline characteristics between the two groups were comparable (*p* > 0.05), enrolling patients with ASA physical status I–III and a mean age ranging from 46.25 ± 8.25 to 73.4 ± 3.1 years. All included studies were conducted in China and focused on airway interventions including bronchoscopy, flexible bronchoscopy, and fiberoptic bronchoscopy. The SJOV intervention varied by delivery device and parameters. Control groups received conventional oxygen therapy (nasal cannula, 4–12 L/min; nasopharyngeal/oropharyngeal airway oxygen), WNJ oxygenation (4 L/min), or mechanical ventilation via modified laryngeal mask airway. Detailed characteristics of included studies are summarized in Table 1.

**Table 1:** Basic characteristics of the studies.

Study	Country	Study design	Age		Sample size		Intervention		Outcome
			T	C	T	C	T	C	
Wei 2024 (40)	China	RCT 3-arm, Blinded	62 ± 17	59 ± 15	44	44	SJOV via WNJ	Nasal cannula oxygen (4 L/min)	①④⑤⑥⑪⑫⑬⑰⑱
				59 ± 9.9		44	(15 psi, 15 bpm, I:E = 1:2)	WNJ oxygenation (4 L/min)	
Yang 2021 (50)	China	RCT Blinded	54.2 ± 15.5	49.1 ± 14.4	50	50	SJOV via Endoscopic mask (0.1 MPa, 15 bpm, I:E = 1:1.5)	Endoscopic mask oxygen (12 L/min)	①②③⑨⑩⑭⑮⑯⑰
			51.6 ± 14.4		50		SJOV via Endoscopic mask (0.1 MPa, 1,200 bpm, I:E = 1:1.5)		
Zha 2021 (30)	China	RCT Blinded	51.7 ± 13.8	53.4 ± 13.6	137	135	SJOV via WNJ (103kpa, 20 bpm, I:E = 1:2)	Nasal cannula oxygen (4 L/min)	①④⑤⑥⑦⑧⑪⑫⑬⑰⑲⑳
Liang 2023a (45)	China	RCT	68.22 ± 1.47	68.29 ± 1.54	60	60	SJOV via Nasopharyngeal airway (0.08–0.16 MPa, 60–100 bpm, I:E = 1:1.5–1:2)	Nasopharyngeal airway oxygen (4 L/min)	②③⑨⑭⑮⑯
Liang 2023b (46)	China	RCT	65.29 ± 5.02	65.58 ± 5.14	60	60	SJOV via Nasopharyngeal airway	Nasopharyngeal airway oxygen	①②③⑨⑯
Zeng 2023 (51)	China	RCT	46.25 ± 8.25	46.29 ± 2.88	60	60	SJOV via Nasopharyngeal airway Manujet III	Nasopharyngeal airway oxygen	①②⑦⑧⑭⑮⑯⑰⑱⑲⑳
Li 2019 (47)	China	RCT	73.4 ± 3.1	72.9 ± 4.0	30	30	SJOV via WNJ (35–45 psi, 8–20 bpm)	Nasal cannula oxygen (5 L/min)	①③⑦⑧⑨⑭⑮⑯⑰⑱⑲⑳
Wu 2017 (32)	China	RCT	52 ± 18	49 ± 16	40	37	SJOV via Nasopharyngeal airway (22–29 psi, 15–20 bpm)	Mechanical ventilation via Modified laryngeal mask airway	②⑨⑪⑭⑮⑯⑰
Wang 2016 (31)	China	RCT	18 ~ 65	18 ~ 65	14	14	SJOV via WNJ (0.15 MPa, 100 bpm, I:E = 1:2)	Mechanical ventilation via Modified laryngeal mask airway	①②③⑦⑨⑩⑭⑮⑯⑰⑱⑲⑳
Tian 2016 (52)	China	RCT	59 ± 7	57 ± 7	40	40	SJOV via Oropharyngeal airway (0.4 kpa, 60 bpm, I:E = 1:2)	Oropharyngeal airway oxygen (4 L/min)	①②④⑥⑭⑮⑯⑰⑲⑳

T, Test group; C, Control group; SJOV, Supraglottic jet oxygenation and ventilation; WNJ, Wei Nasal Jet^®^ Tube; SpO_2_, Oxygen Saturation; PaO₂, Arterial Oxygen Partial Pressure; PaCO₂, Partial Pressure of Carbon Dioxide; Endpoint indicators: ① incidence of hypoxemia; ② Intraoperative SpO_2_; ③ Intraoperative PaO₂; ④ Jaw Lift; ⑤ Adjustment of Ventilator Parameters; ⑥ Mask Ventilation with Positive Pressure; ⑦ Tachycardia; ⑧ Hypertension; ⑨ Intraoperative PaCO₂; ⑩ Intraoperative Potential of hydrogen; ⑪ Sore Throat; ⑫ Xerostomia; ⑬ Nasal Bleeding; ⑭ Heart Rate; ⑮ Mean Arterial Pressure; ⑯ SpO₂; ⑰ Procedure Time; ⑱ Recovery Time; ⑲ Physician Satisfaction; ⑳ Patient Satisfaction.

### 3.2 Risks of bias

**Figure 2:**
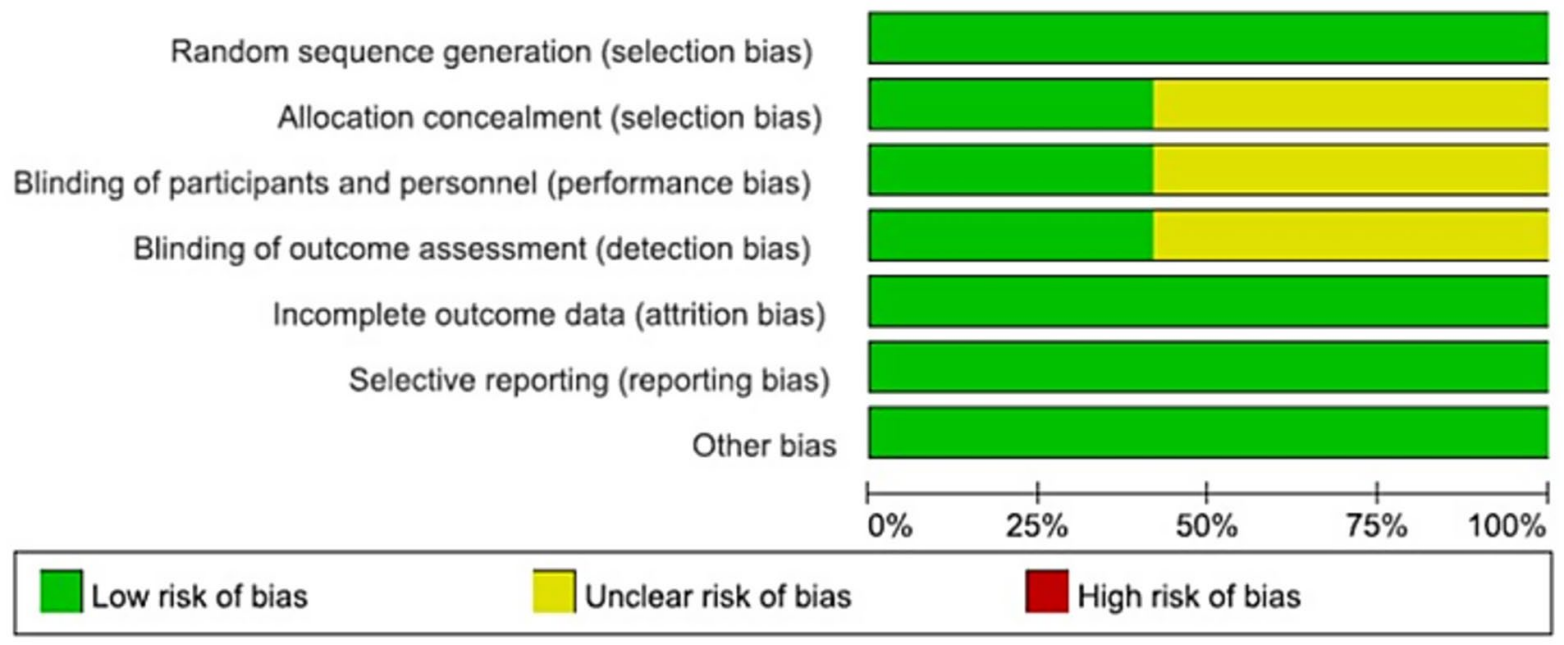
Risk of bias graph.

**Figure 3:**
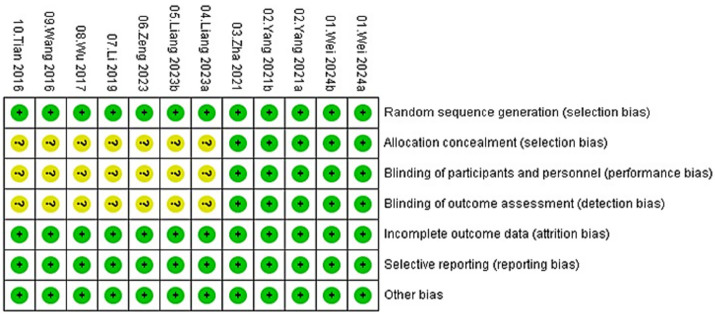
Risk of bias summary.

Overall, the majority of studies exhibited low risk of bias in domains related to incomplete outcome data and selective reporting, with most studies providing adequate follow-up and reporting protocols. Specifically, all 10 studies were judged to have low risk in selective reporting and incomplete outcome data. For random sequence generation, 7 studies (70%) were judged as low risk, while the remaining 3 studies had unclear risk due to insufficient detail regarding the randomization method. Allocation concealment, blinding of participants and personnel, and blinding of outcome assessment were consistently rated as unclear in all 10 studies due to insufficient reporting. None of the trials provided details on allocation concealment, blinding procedures for participants/personnel, or blinding of outcome assessors, resulting in an unclear risk of selection, performance, and detection bias across these domains. Other bias was judged as unclear in all studies due to lack of information on potential confounding factors (Figures 2, 3).

### 3.3 Primary outcome: incidence of intraoperative hypoxemia, intraoperative oxygen saturation (SpO₂) and intraoperative arterial oxygen partial pressure (PaO₂)

#### 3.3.1 Incidence of intraoperative hypoxemia

A total of 8 studies were included in the meta-analysis of hypoxemia incidence, involving 529 patients in the experimental group and 527 patients in the control group, and demonstrated substantial heterogeneity (*I*^2^ = 80%, *p* < 0.00001). Thus, random-effects model (Mantel–Haenszel method) showed that the experimental group had a significantly lower incidence of hypoxemia compared with the control group (OR = 0.20, 95% CI: 0.07–0.58).

#### 3.3.2 Intraoperative SpO₂

Eight studies (including subgroups) reported intraoperative SpO₂ at fixed intraoperative time points, with 374 patients in the experimental group and 371 patients in the control group, and demonstrated substantial heterogeneity (*I*^2^ = 99%, *p* < 0.00001). Thus, random-effects model demonstrated that the experimental group had a significantly higher mean intraoperative SpO₂ compared with the control group (MD = 2.27, 95% CI: 0.12–4.42).

#### 3.3.3 Intraoperative PaO₂

**Figure 4:**
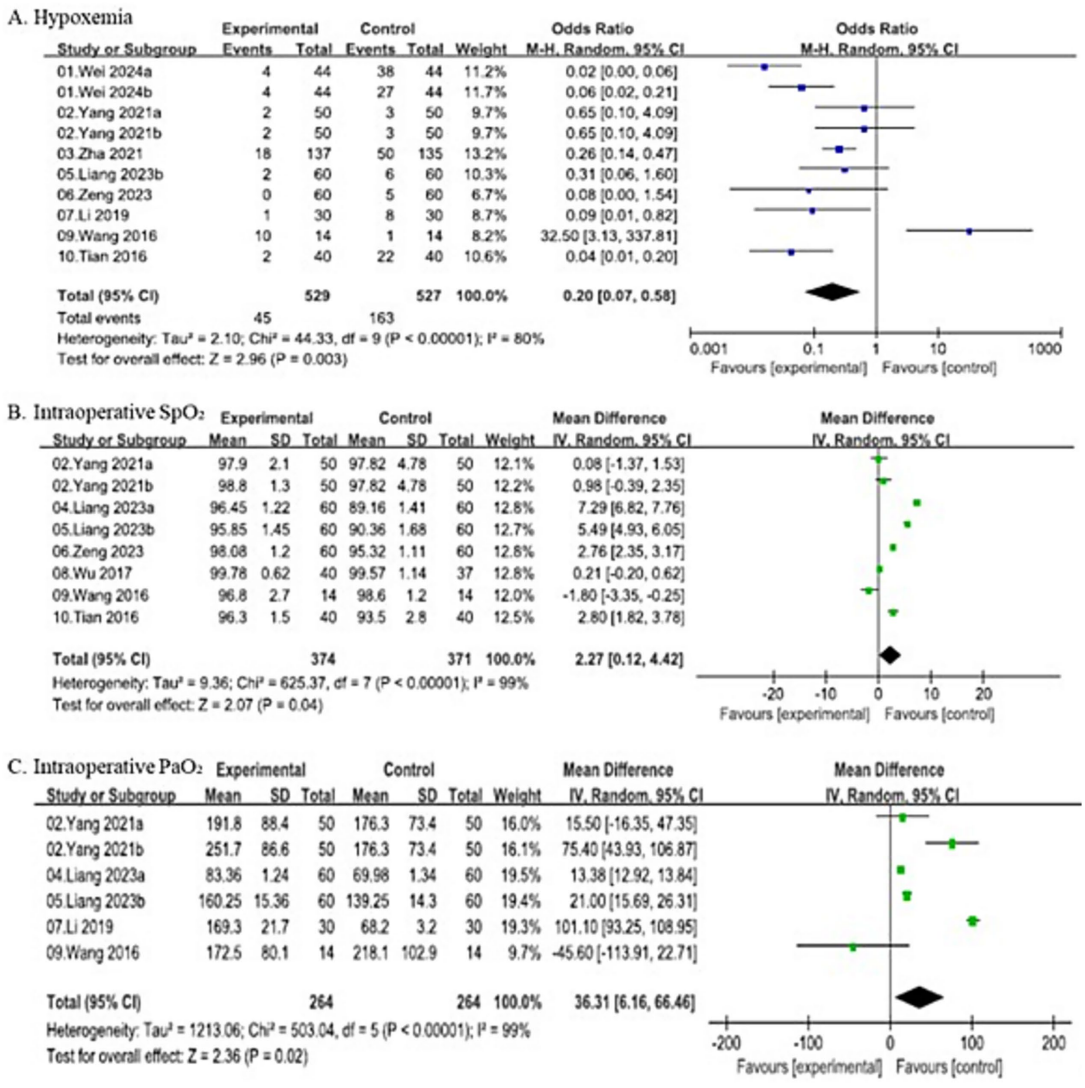
Forest plots comparing primary outcome between SJOV group and control group.

PaO₂ values were also collected at fixed intraoperative time points as defined in each trial. Six studies (including subgroups) provided data on PaO₂, involving 264 patients in both the experimental and control groups, and demonstrated substantial heterogeneity (*I*^2^ = 99%, *p* < 0.00001). Thus, random-effects model revealed that the experimental group had a significantly higher mean PaO₂ compared with the control group (MD = 36.31, 95% CI: 6.16–66.46). All the details are shown in Figure 4.

#### 3.3.4 Subgroup and sensitivity analyses of primary outcomes

##### 3.3.4.1 Subgroup analyses based on control-group oxygenation strategy

Subgroup analyses stratified by control group oxygenation strategy demonstrated that the overall reduction in intraoperative hypoxemia associated with the experimental intervention was primarily driven by comparisons with conventional oxygen strategies. In this subgroup, the experimental intervention was associated with a significantly lower incidence of hypoxemia (OR = 0.17, 95% CI: 0.04–0.65). In contrast, when compared with advanced ventilation strategies, the reduction in hypoxemia incidence was attenuated and did not reach statistical significance (OR = 0.27, 95% CI: 0.03–2.16).

For oxygenation related continuous outcomes, similar patterns were observed. Improvements in intraoperative SpO₂ and PaO₂ were more pronounced when the control group received conventional oxygen strategies, whereas smaller and more heterogeneous effects were observed when advanced ventilation strategies were used as comparators. Tests for subgroup differences did not demonstrate statistically significant interaction effects, indicating that the observed differences should be interpreted cautiously.

##### 3.3.4.2 Subgroup analyses based on SJOV driving pressure

In subgroup analyses stratified by SJOV driving pressure, a statistically significant reduction in hypoxemia incidence was observed in the low-pressure subgroup, whereas effect estimates in the high-pressure subgroup was highly variable, with wide confidence intervals and inconsistent effect directions, precluding definitive conclusions. Studies with unreported pressure parameters (NR) also showed a reduction in hypoxemia incidence, although the number of contributing trials was limited.

For continuous oxygenation outcomes, pressure-based subgroup analyses of intraoperative SpO₂ and PaO₂ demonstrated heterogeneous and inconsistent effects across pressure strata, with substantial within subgroup heterogeneity and overlapping confidence intervals. No clear or consistent improvement in SpO₂ or PaO₂ could be identified for either pressure category.

Given the incomplete reporting of pressure parameters in some trials, sensitivity analyses excluding studies with unreported pressure values were performed. After exclusion of these studies, the pooled odds ratio for hypoxemia incidence was attenuated and no longer reached statistical significance, with widened confidence intervals indicating reduced precision of the effect estimate. Sensitivity analyses for intraoperative SpO₂ and PaO₂ similarly showed attenuated and non-significant pooled effects, without a consistent pattern of benefit after exclusion of studies with incomplete pressure reporting.

Overall, pressure-based subgroup and sensitivity analyses did not identify a clear pressure threshold associated with consistent improvements across hypoxemia incidence, SpO₂, and PaO₂.

##### 3.3.4.3 Subgroup analyses based on SJOV driving frequency

Subgroup analyses stratified by SJOV driving frequency demonstrated a differential pattern of effects on intraoperative hypoxemia. A significant reduction in hypoxemia incidence was observed in the low-frequency subgroup, whereas the pooled effect in the high-frequency subgroup was not statistically significant, with wide confidence intervals and substantial heterogeneity, indicating an unstable effect estimate. Studies with unreported driving frequency (NR) also showed a reduction in hypoxemia incidence, although the number of contributing trials was limited.

For continuous oxygenation outcomes, frequency-based subgroup analyses of intraoperative SpO₂ showed no significant difference in the low-frequency subgroup, while the high-frequency subgroup demonstrated a non-significant trend toward higher SpO₂ with considerable heterogeneity. Similarly, subgroup analyses of intraoperative PaO₂ revealed no statistically significant differences in either low or high frequency strata, with wide confidence intervals and marked between study variability.

To further assess the robustness of these findings, sensitivity analyses excluding studies with unreported driving frequency were performed. After exclusion of NR studies, the overall pooled effect for hypoxemia incidence remained in the same direction and statistically significant, whereas pooled effects for SpO₂ and PaO₂ were attenuated and no longer statistically significant, reflecting reduced precision of the estimates.

Overall, frequency based subgroup and sensitivity analyses suggest that the reduction in hypoxemia associated with SJOV is primarily driven by studies using lower driving frequencies, while evidence for frequency-dependent effects on SpO₂ and PaO₂ remains inconclusive.

### 3.4 Secondary outcome: incidence of airway interventions for hypoxemia, arteria blood gas, peri-operative adverse events

#### 3.4.1 Incidence of airway interventions for hypoxemia

Three airway interventions performed for hypoxemia (jaw lift, adjustment of ventilator parameters, and mask ventilation with positive pressure) were analyzed as secondary outcomes, with results consistently demonstrating a significant reduction in intervention requirements in the SJOV group compared to the control group.

##### 3.4.1.1 Jaw lift

A total of 4 studies involving 528 patients (265 in the experimental group and 263 in the control group) reported the incidence of jaw lift due to hypoxemia, and demonstrated substantial heterogeneity (*I*^2^ = 79%, *p* = 0.002). Thus, random-effects model showed that the SJOV group had a significantly lower risk of requiring jaw lift compared to the control group (OR = 0.09, 95% CI: 0.03–0.30).

After excluding Zha (30), 3 studies involving 256 patients (128 in the experimental group and 128 in the control group) were included. The fixed-effects model showed that the SJOV group still had a significantly lower risk of requiring jaw lift compared to the control group (OR = 0.06, 95% CI: 0.03–0.11).

##### 3.4.1.2 Adjustment of ventilator parameters

Three studies involving 448 patients (225 in the SJOV group and 223 in the control group) evaluated the need for adjusting ventilator parameters (oxygen flow rate, driving pressure) to correct hypoxemia. The fixed-effects model revealed a significant reduction in parameter adjustment requirements in the SJOV group (OR = 0.05, 95% CI: 0.03–0.12, *I*^2^ = 45%). Total events of parameter adjustment were 12 in the SJOV group and 82 in the control group.

##### 3.4.1.3 Mask ventilation with positive pressure

**Figure 5:**
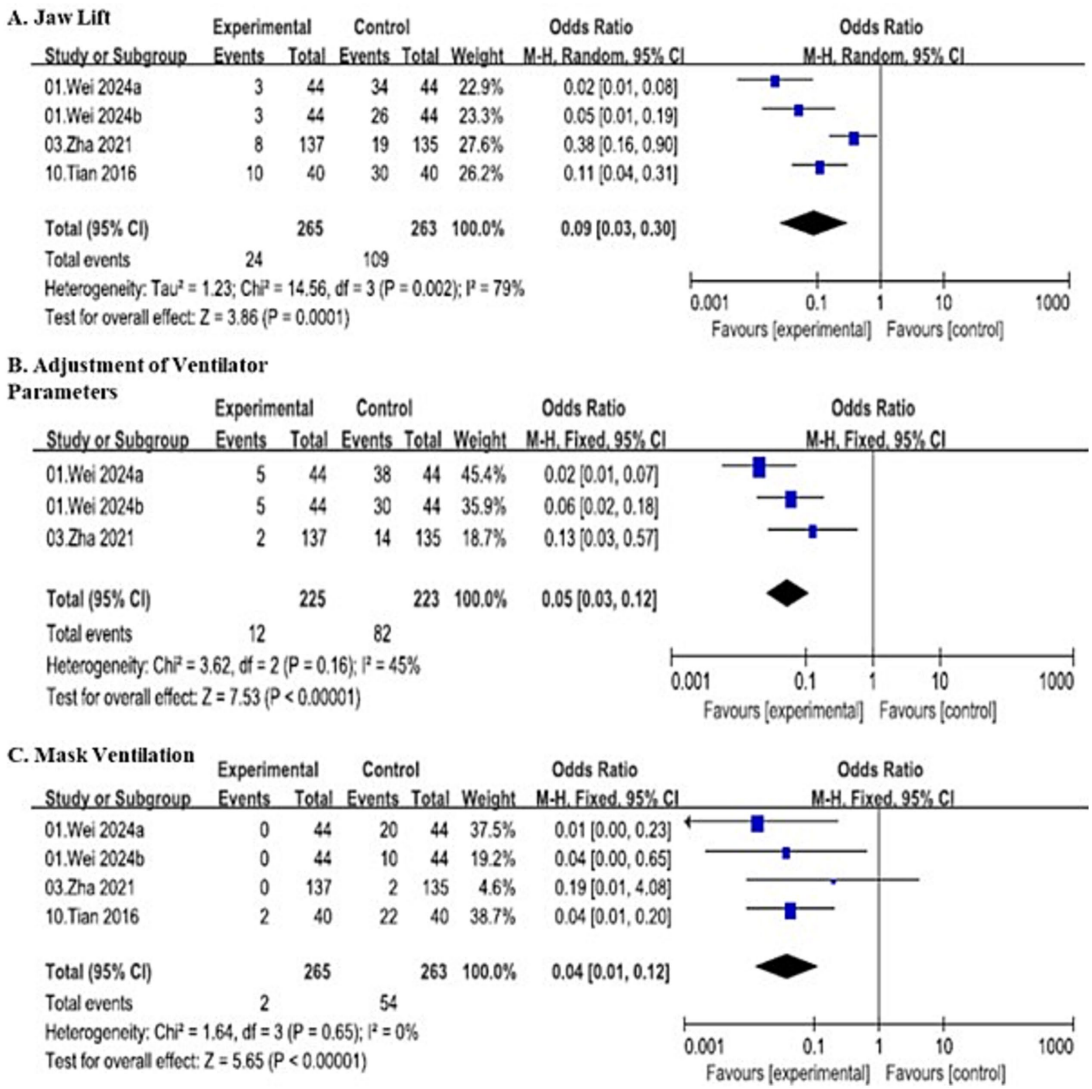
Forest plots comparing incidence of airway interventions for hypoxemia between SJOV group and control group.

Four studies involving 528 patients (265 in the SJOV group and 263 in the control group) reported the incidence of mask ventilation with positive pressure to resolve hypoxemia. The fixed-effects model demonstrated a marked reduction in this intervention in the SJOV group (OR = 0.04, 95% CI: 0.01–0.12, *I*^2^ = 0%). Total events of mask ventilation were 2 in the SJOV group and 54 in the control group. All the details are shown in Figure 5.

#### 3.4.2 Intra-operative adverse events

##### 3.4.2.1 Tachycardia

A total of 4 studies were initially included to evaluate intraoperative tachycardia, involving 480 patients (241 in the SJOV group and 239 in the control group), and demonstrated substantial heterogeneity (*I*^2^ = 69%, *p* = 0.02). Thus, random-effects model showed no significant difference in tachycardia incidence between the two groups (OR = 0.77, 95% CI: 0.13–4.56), with 13 events in the SJOV group and 17 in the control group.

A sensitivity analysis excluding Wang (31) was performed, leaving 3 studies with 452 patients (227 in the SJOV group and 225 in the control group). The fixed-effects model revealed that the SJOV group had a significantly lower incidence of tachycardia (OR = 0.35, 95% CI: 0.14–0.91, *I*^2^ = 15%), with 6 events in the SJOV group and 16 in the control group.

##### 3.4.2.2 Hypertension

**Figure 6:**
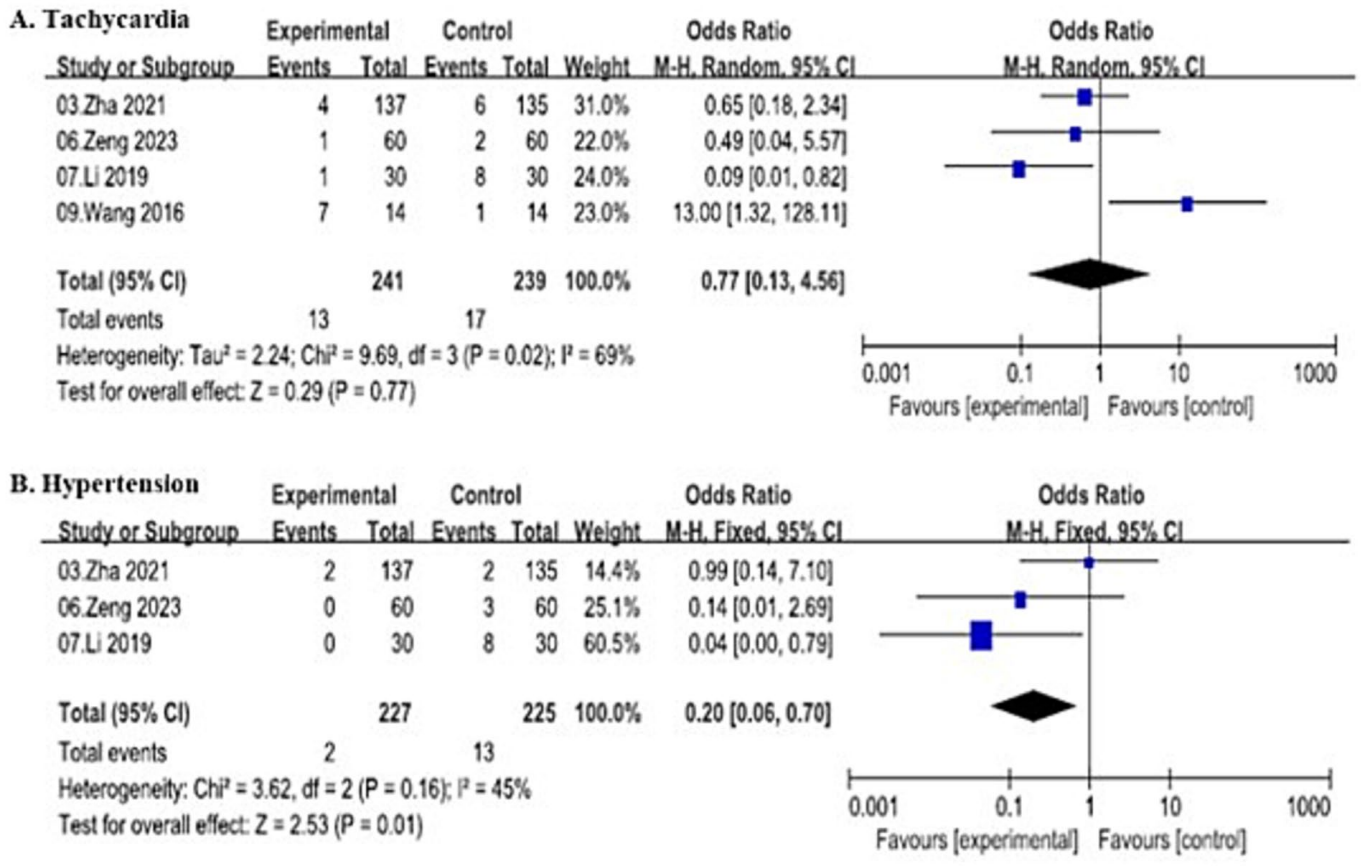
Forest plots comparing intra-operative adverse events between SJOV group and control group.

Three studies involving 452 patients (227 in the SJOV group and 225 in the control group) reported intraoperative hypertension. The fixed-effects model demonstrated a significantly lower incidence of hypertension in the SJOV group (OR = 0.20, 95% CI: 0.06–0.70, *I*^2^ = 45%), with 2 events in the SJOV group and 13 in the control group. All the details are shown in Figure 6.

#### 3.4.3 Intra-operative arteria blood gas

##### 3.4.3.1 Potential of hydrogen

Three studies involving 228 patients (114 in the SJOV group and 114 in the control group) evaluated intraoperative pH levels, and demonstrated substantial heterogeneity (*I*^2^ = 93%, *p* < 0.00001). Thus, random-effects model demonstrated no significant difference in pH between the two groups (MD = −0.04, 95% CI: −0.12–0.04).

##### 3.4.3.2 Partial pressure of carbon dioxide

**Figure 7:**
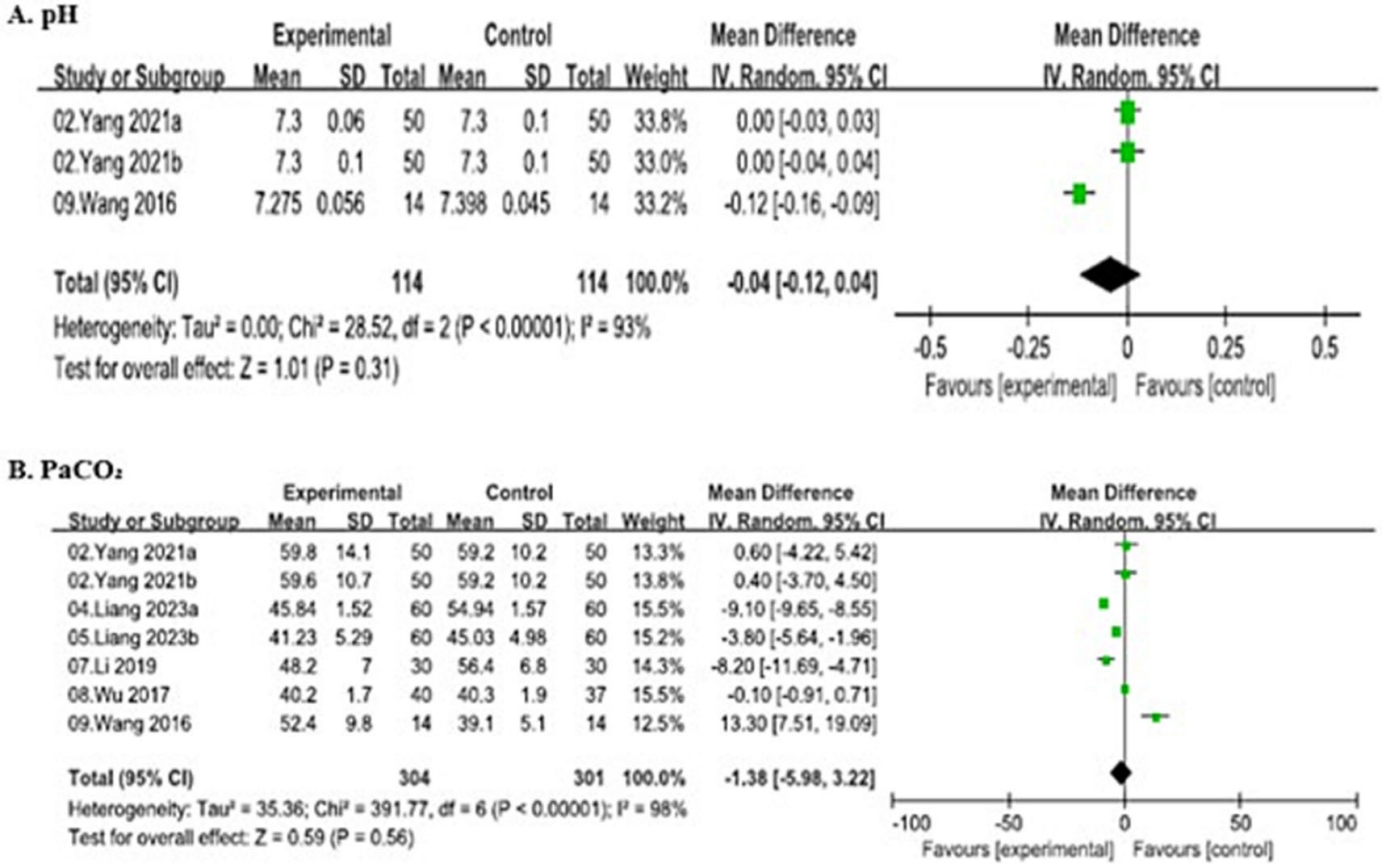
Forest plots comparing intra-operative arteria blood gas between SJOV group and control group.

Seven studies involving 605 patients (301 in the SJOV group and 304 in the control group) reported intraoperative PaCO₂ and demonstrated substantial heterogeneity (*I*^2^ = 98%, *p* < 0.00001). Thus, random-effects model showed no significant difference in PaCO₂ between the two groups (MD = −1.38, 95% CI: −5.98–3.22). All the details are shown in Figure 7.

#### 3.4.4 Post-operative adverse events

##### 3.4.4.1 Sore throat

A total of 4 studies (including subgroups) were initially included to analyze the incidence of post-procedural sore throat, involving 525 patients (265 in the SJOV group and 260 in the control group) and demonstrated substantial heterogeneity (*I*^2^ = 91%, *p* < 0.00001). Thus, random-effects model showed no significant difference in sore throat incidence between the two groups (OR = 0.68, 95% CI: 0.15–3.17), with 65 events in the SJOV group and 70 in the control group.

After excluding Wu (32), 3 studies involving 448 patients (225 in the SJOV group and 223 in the control group) were included. The fixed-effects model revealed that the SJOV group had a significantly higher incidence of sore throat (OR = 1.71, 95% CI: 1.08–2.71, *I*^2^ = 0%), with 60 events in the SJOV group and 40 in the control group.

##### 3.4.4.2 Xerostomia

Three studies involving 448 patients (225 in the SJOV group and 223 in the control group) reported the incidence of post-procedural xerostomia. The fixed-effects model demonstrated a significantly higher incidence of xerostomia in the SJOV group (OR = 6.08, 95% CI: 2.99–12.36, *I*^2^ = 44%), with 50 events in the SJOV group and 11 in the control group.

##### 3.4.4.3 Nasal bleeding

**Figure 8:**
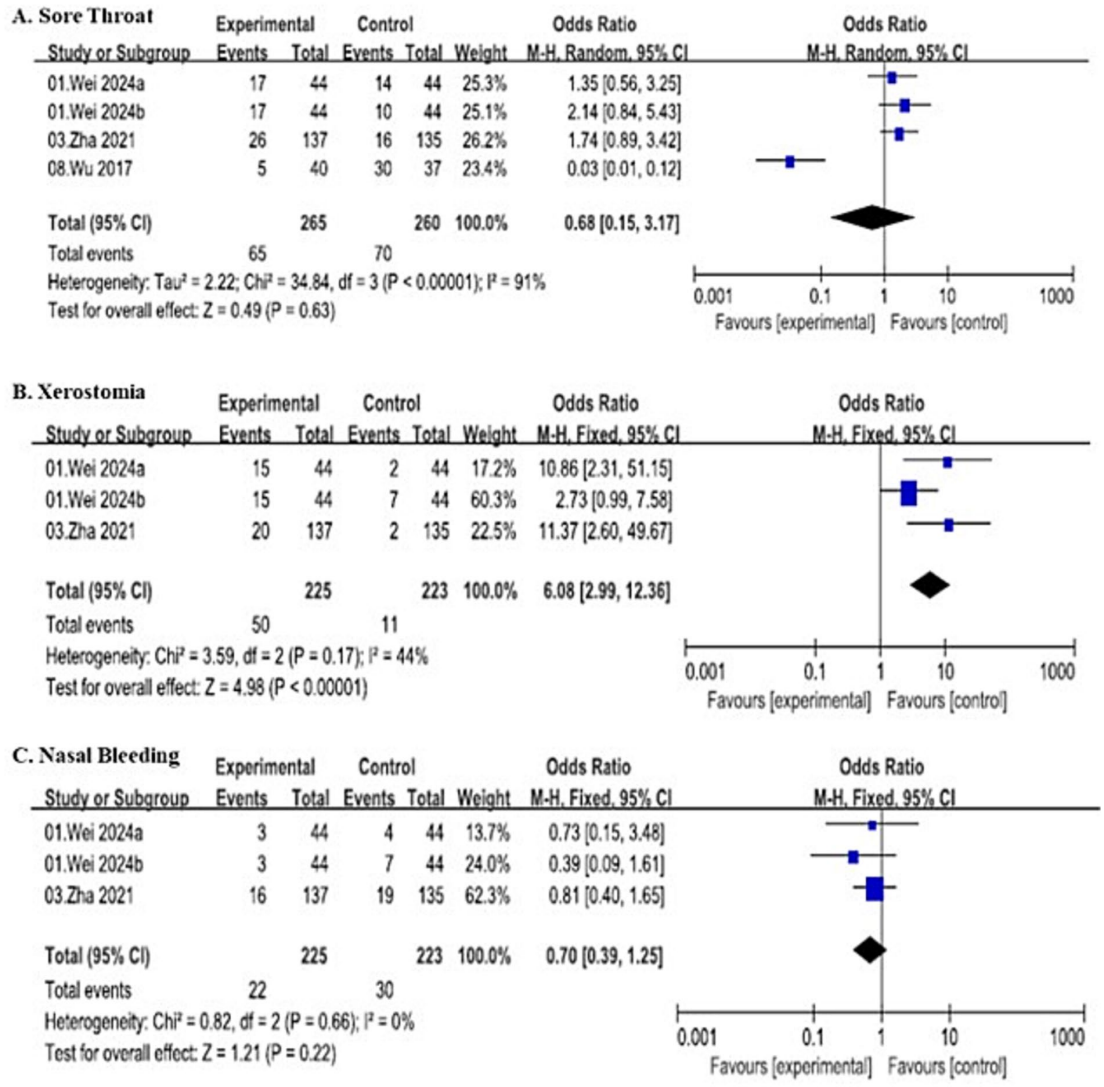
Forest plots comparing post-operative adverse events between SJOV group and control group.

Three studies involving 448 patients (225 in the SJOV group and 223 in the control group) evaluated post-procedural nasal bleeding. The fixed-effects model showed no significant difference in nasal bleeding incidence between the two groups (OR = 0.70, 95% CI: 0.39–1.25, *I*^2^ = 0%), with 22 events in the SJOV group and 30 in the control group. All the details are shown in Figure 8.

### 3.5 Other secondary outcomes

#### 3.5.1 Hemodynamic parameters

##### 3.5.1.1 Heart rate

Eight studies reported HR data at four time points (pre-operative: T_1_, post-induction: T_2_, intra-operative: T_3_, and post-operative: T_4_), involving 685 patients (344 in the SJOV group and 341 in the control group).

The fixed-effects model showed a small but statistically significant increase in HR at T_1_ in the SJOV group compared to the control group (MD = 1.18, 95%CI: 0.12–2.24, *I*^2^ = 0%). No significant difference in HR at T_2_–T_4_ was observed between groups and marked heterogeneity was noted (MD = 0.05, 95% CI: −2.61–2.72, *I*^2^ = 76%; MD = 0.92, 95% CI: −2.57–4.41, *I*^2^ = 87%; MD = 1.57, 95% CI: −1.81–4.94, *I*^2^ = 85%) using the random-effects model.

##### 3.5.1.2 Mean arterial pressure

Eight studies evaluated MAP at four time points, involving 685 patients (344 in the SJOV group and 341 in the control group). No significant difference was observed in MAP at T_1_–T_4_ (MD = 0.18, 95% CI: −0.27–0.62; MD = −6.36, 95% CI: −14.40–1.68; MD = −4.61, 95% CI: −10.39–1.17; MD = −2.13, 95% CI: −5.52–1.25) using the random-effects model, with extreme heterogeneity (*I*^2^ = 44%; *I*^2^ = 99%; *I*^2^ = 98%; *I*^2^ = 94%).

##### 3.5.1.3 SpO₂

Nine studies reported SpO₂ at T_1_, and eight studies at T_2_–T_4_, involving 805 patients (404 in the SJOV group and 401 in the control group) at T_1_, and 745 patients (374 in the SJOV group and 371 in the control group) at T_2_–T_4_. No significant difference was observed in T_1_, T_2_, and T_4_ (MD = −0.17, 95% CI: −0.67–0.33; MD = 1.32, 95% CI: −0.48–3.11; MD = 1.82, 95% CI: −0.11–3.74) using the random-effects model, with extreme heterogeneity (*I*^2^ = 89%; *I*^2^ = 99%; *I*^2^ = 99%). The random-effects model demonstrated a small but statistically significant increase in SpO₂ at T_3_ in the SJOV group (MD = 2.27, 95% CI: 0.12–4.42) with extreme heterogeneity (*I*^2^ = 99%). All the details are shown in Table 2.

**Table 2:** Characteristics of intra-operative hemodynamic parameters.

Characteristics	Study number	Total patients (T + C)	MD	95%CI	*P*	Tau^2^	Chi^2^	df	*I* ^2^
HR-T_1_	8	685 (344 + 341)	1.18	[0.12, 2.24]	0.03	–	6.83	7	0%
HR-T_2_	8	685 (344 + 341)	0.05	[−2.61, 2.72]	0.97	8.44	29.34	7	76%
HR-T_3_	8	685 (344 + 341)	0.92	[−2.57, 4.41]	0.60	17.77	53.75	7	87%
HR-T_4_	8	685 (344 + 341)	1.57	[−1.81, 4.94]	0.36	16.46	47.62	7	85%
MAP-T_1_	8	685 (344 + 341)	0.18	[−0.27, 0.62]	0.44	–	12.39	7	44%
MAP-T_2_	8	685 (344 + 341)	6.36	[−14.40, 1.68]	0.12	125.14	810.12	7	99%
MAP-T_3_	8	685 (344 + 341)	4.61	[−10.39, 1.17]	0.12	62.64	337.21	7	98%
MAP-T_4_	8	685 (344 + 341)	2.13	[−5.52, 1.25]	0.22	17.67	115.64	7	94%
SpO_2_-T_1_	9	805 (404 + 401)	0.17	[−0.67, 0.33]	0.50	0.46	73.96	8	89%
SpO_2_-T_2_	8	745 (374 + 371)	1.32	[−0.48, 3.11]	0.15	6.55	672.40	7	99%
SpO_2_-T_3_	8	745 (374 + 371)	2.27	[0.12, 4.42]	0.04	9.36	625.37	7	99%
SpO_2_-T_4_	8	745 (374 + 371)	1.82	[−0.11, 3.74]	0.06	7.35	1169.38	7	99%

T_1_, Pre-operative, T_2_, Post-induction, T_3_, Intra-operative, T_4_, Post-operative; HR, Heart Rate; MAP, Mean Arterial Pressure; SpO_2_, Oxygen Saturation.

#### 3.5.2 Procedure characteristics

##### 3.5.2.1 Procedure time

Ten studies involving 1,013 patients (509 in the SJOV group and 504 in the control group) reported total procedure time and demonstrated substantial heterogeneity (*I*^2^ = 91%, *p* < 0.00001). Thus, random-effects model showed a statistically significant reduction in procedure time in the SJOV group compared to the control group (MD = −2.80, 95% CI: −5.56–-0.03).

##### 3.5.2.2 Recovery time

Five studies involving 384 patients (192 in the SJOV group and 192 in the control group) evaluated post-procedural recovery time and demonstrated substantial heterogeneity (*I*^2^ = 98%, *p* < 0.00001). Thus, random-effects model demonstrated a significantly shorter recovery time in the SJOV group (MD = −2.90, 95% CI: −5.49–-0.30).

##### 3.5.2.3 Physician satisfaction

Five studies involving 560 patients (281 in the SJOV group and 279 in the control group) assessed physician satisfaction using standardized scales and demonstrated substantial heterogeneity (*I*^2^ = 95%, *p* < 0.00001). Thus, random-effects model showed no significant difference in physician satisfaction between groups (MD = 0.34, 95% CI: −0.65–1.32).

##### 3.5.2.4 Patient satisfaction

**Figure 9:**
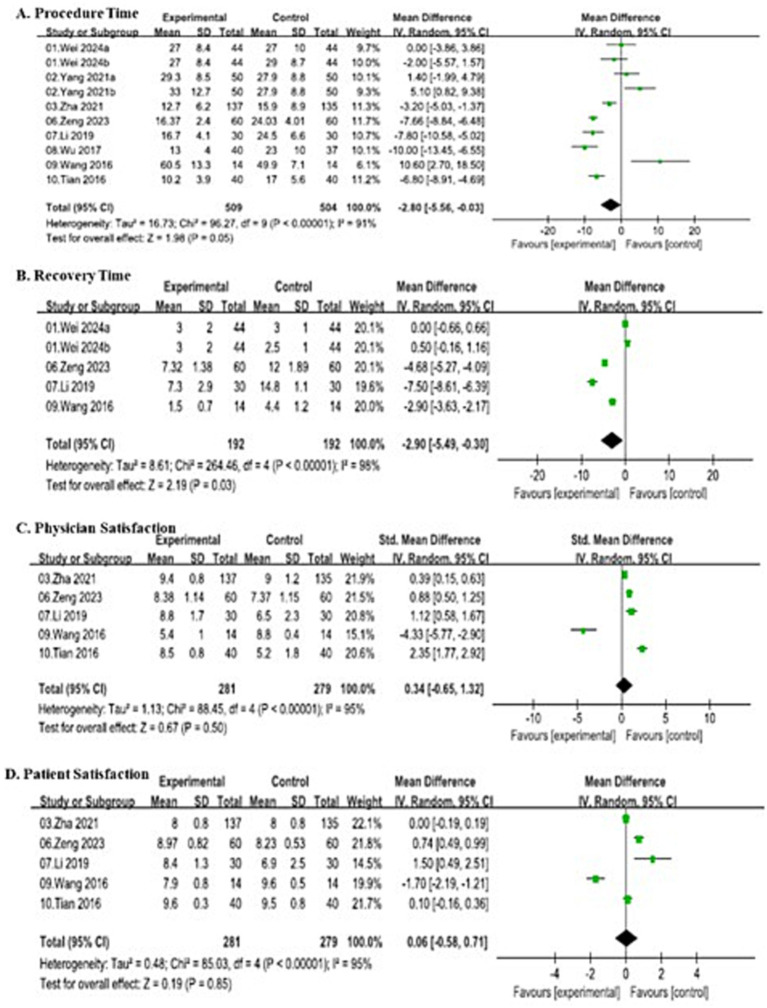
Forest plots comparing procedure characteristics between SJOV group and control group.

Five studies involving 560 patients (281 in the SJOV group and 279 in the control group) evaluated patient satisfaction and demonstrated substantial heterogeneity (*I*^2^ = 95%, *p* < 0.00001). Thus, random-effects model showed no significant difference between groups (MD = 0.06, 95% CI: −0.58–0.71). All the details are shown in Figure 9.

### 3.6 Grade evidence certainty

As assessed by the GRADE approach, the certainty of evidence for 13 outcomes was uniformly rated as low (⨁⨁◯◯). This downgrading was primarily driven by two serious concerns: serious inconsistency and serious imprecision, with no other critical issues identified. Although sensitivity analyses confirmed the robustness of pooled results, removing individual studies one at a time did not meaningfully alter effect sizes or directions, and no single study exerted undue influence. The unidentifiable source of moderate to high heterogeneity in certain outcomes further contributed to the low evidence certainty. Thus, while the findings are statistically stable, they should be interpreted with caution in clinical practice, awaiting additional high-quality studies to address existing limitations.

## 4 Discussion

### 4.1 Core findings

This meta-analysis of 10 RCTs that SJOV significantly improves patient safety and procedural efficiency during bronchoscopic procedures. The primary finding indicates that SJOV markedly reduced the incidence of intraoperative hypoxemia and significantly enhanced intraoperative oxygenation levels. Consequently, the requirement for rescue airway interventions, including jaw lift adjustment of ventilator parameters, and positive-pressure mask ventilation, was drastically reduced in the SJOV group.

### 4.2 Efficacy and mechanisms of SJOV

The most important feature of the supraglottic airway is its tendency to collapse significantly after anesthesia, which results in a narrower airway and makes it difficult for airflow to pass through the constricted region (33, 34). The high-speed airflow generated by SJOV can effectively wash out the dead space of the pharyngeal cavity, generate functional PEEP, and markedly reduce the impact of airway collapse and mechanical obstruction. Furthermore, the high-velocity oxygen jets generate functional PEEP, which helps maintain airway patency and prevent alveolar collapse, particularly crucial when the bronchoscope occupies the airway and exacerbates ventilation-perfusion mismatch (28, 35, 36). This dual mechanism explains the significant reduction in hypoxemia incidence and the consistent improvement in oxygenation parameters observed across studies.

The magnitude of clinical benefit was most pronounced in high-risk populations, such as elderly patients or those with underlying obese, obstructive sleep apnea (OSA), COPD, difficult airway, suggesting that SJOV may be particularly valuable in patients with diminished respiratory reserve (27, 37). A previous study had reported hypoxemia rates as high as 91% in patients receiving nasal cannula oxygen during bronchoscopy (38), primarily due to inadequate oxygen diffusion when the airway is partially or fully obstructed by the instrument, and participants suffering from COPD and classified as ASA Class III or higher (13, 39). The superior efficacy of SJOV in preventing hypoxemia stems from its ability to overcome the fundamental limitations of conventional oxygen therapy during bronchoscopy (40, 41).

In this study, compared with other oxygen therapies, SJOV halved the incidence of intraoperative hypoxemia, with a pooled OR of 0.20. Across the 10 included studies, the total number of hypoxemia events was 45 in the SJOV group versus 163 in the control group, highlighting a clear protective effect of SJOV against hypoxemia. Another meta-analysis demonstrates that SJOV significantly reduces the risk of hypoxemia and severe hypoxemia during procedural sedation (RR = 0.26, and 0.22) (36).

### 4.3 Oxygenation improvement and clinical implications

A critical advantage highlighted in guidelines noting that pulse oximetry alone monitoring often misses hypoventilation in sedated patients (42). Intraoperative blood gas PaO_2_ and continuous SpO_2_ monitoring are critical. SJOV significantly enhanced intraoperative oxygenation, as evidenced by marked improvements in both continuous monitoring parameters and arterial blood gas values. The pooled analysis demonstrated a mean SpO₂ increase of 2.27% and a PaO₂ increase of 36.31 mmHg in SJOV treated patients. The improvements that substantially exceed the typical 10–20 mmHg decline in PaO₂ observed during bronchoscopy with conventional oxygenation (43, 44). The most substantial benefit occurred in trials utilizing nasopharyngeal airway delivery systems (45, 46) and in studies focusing on high-risk populations (47), suggesting that both device selection and patient selection influence the magnitude of effect.

The physiological basis for this enhancement lies in SJOV’s ability to deliver 100% oxygen directly to the supraglottic space via devices such as the WNJ tube or nasopharyngeal airway. This creates a continuous airflow barrier that minimizes room air dilution, flushes anatomical dead space, and generates functional positive end-expiratory pressure, thereby maintaining functional residual capacity and preventing end-expiratory alveolar collapse even with bronchoscope-induced airway obstruction.

Notably, high heterogeneity was observed across these outcomes, attributable to variations in SJOV parameters, patient characteristics, procedural interventions, and measurement timing. Rather than undermining the results, this variability underscores the robustness of the oxygenation benefit across diverse clinical scenarios and suggests that the effect size may be modulated by technical and patient-related factors. This observation indicates that the benefits of SJOV are generalizable, but also highlights the need for future studies to identify the optimal parameters for specific patient subgroups.

To further explore the sources of heterogeneity, subgroup and sensitivity analyses were conducted based on control-group oxygenation strategies and SJOV technical parameters. These analyses suggested that the magnitude of oxygenation benefit was influenced by comparator intensity, with more pronounced effects observed when SJOV was compared with conventional oxygen therapy. Exploratory analyses based on driving pressure and frequency did not identify consistent parameter thresholds associated with superior outcomes, and several subgroup findings were not robust in sensitivity analyses excluding studies with unreported parameters. These results indicate that subgroup analyses are hypothesis-generating and primarily serve to contextualize heterogeneity rather than to define optimal SJOV settings.

### 4.4 Reduction in rescue interventions and procedural efficiency

The robust oxygenation provided by SJOV translated into significantly reduced need for rescue interventions (26). The requirement for jaw lift, ventilator parameter adjustment, and positive-pressure mask ventilation was reduced by 65–96% in the SJOV group compared to controls. The consistency of this benefit across different rescue modalities, with particularly low heterogeneity for mask ventilation, indicates that SJOV provides reliable procedural stability regardless of operator experience or case complexity.

This reduction in rescue interventions likely contributed to the shorter procedure and recovery times observed in the SJOV group. These time savings result directly from reduced procedural interruptions, a consequence of the more stable oxygenation provided by SJOV.

### 4.5 Safety profile, tolerability, and disadvantages of SJOV

The superior efficacy of SJOV must be evaluated in conjunction with its distinct safety and tolerability profile. Analysis of intra-operative adverse events revealed a reassuring hemodynamic profile. Notably, the incidence of intra-operative hypertension was significantly lower in the SJOV group, a finding that may be attributed to the reduced occurrence of hypoxemia, which is a potent trigger for sympathetic activation and blood pressure elevation. Although a transient increase in pre-operative heart rate was occasionally observed, potentially attributable to device placement or patient anxiety. No clinically significant alterations in blood pressure or sustained tachycardia occurred during or after procedures.

The most common complaints were post-procedural xerostomia and sore throat, likely resulting from the mechanical and desiccating effects of high-flow gas on the oropharyngeal mucosa. Importantly, these symptoms were generally mild and self-limiting, did not diminish overall satisfaction, and stood in contrast to the absence of significant differences in more serious adverse events such as nasal bleeding. Notably, no cases of barotrauma were reported in any included studies, alleviating theoretical concerns regarding high-pressure ventilation (48, 49). However, it should be acknowledged that the relatively small sample sizes and limited follow-up durations of the included trials restrict the ability to detect rare but potentially serious complications.

In addition, SJOV parameter settings, including driving pressure and frequency, varied substantially across studies, and no standardized or consensus-based parameter protocols were applied. This lack of standardization represents a practical limitation of SJOV and may contribute to variability in both efficacy and safety outcomes across clinical settings.

Taken together, the available evidence suggests that SJOV is associated with an acceptable safety and tolerability profile when used during bronchoscopy. While these findings support its potential role as an alternative oxygenation strategy, particularly in patients at high risk of hypoxemia, such as those with chronic obstructive pulmonary disease or significant airway stenosis. Careful patient selection, appropriate monitoring, and operator expertise remain essential.

### 4.6 Clinical heterogeneity related to patient and procedural factors

Patient-related factors, such as underlying pulmonary disease, baseline respiratory reserve, and indication for bronchoscopy, as well as procedural characteristics, such as diagnostic versus therapeutic procedures and procedural duration, are known to influence intraoperative oxygenation. However, in the present meta-analysis, most included trials did not report patient etiologies or procedure subtypes in sufficient detail, precluding reliable subgroup or meta-regression analyses based on these factors.

Procedural duration was the only procedural variable consistently reported across studies and was therefore analyzed as a secondary outcome. Nevertheless, procedure time alone may not fully capture the complexity of procedural heterogeneity, and residual confounding related to unmeasured patient and procedural characteristics may have contributed to the observed heterogeneity. In addition to clinical heterogeneity, methodological factors such as unclear allocation concealment, lack of blinding, and variability in outcome measurement timing may have further contributed to between-study heterogeneity, as reflected in the subgroup and sensitivity analyses.

### 4.7 Limitations and generalizability

Several limitations should be considered when interpreting these findings. A major limitation of the current evidence base is that allocation concealment and blinding of participants, personnel, and outcome assessors were rated as unclear in all included trials due to insufficient reporting. Although outcomes such as hypoxemia incidence and oxygenation parameters are relatively objective, the lack of clear blinding may still introduce performance and detection bias, potentially leading to overestimation of treatment effects, particularly for intraoperative management decisions and rescue interventions.

All included trials were conducted in China, potentially limiting generalizability to other populations. The absence of blinding in most studies introduces potential for performance and detection bias. Moreover, subgroup and sensitivity analyses were exploratory in nature and based on a limited number of studies, and thus should be interpreted cautiously. Significant heterogeneity was observed for many outcomes, reflecting variations in technical parameters, patient characteristics, and measurement timing. Finally, longer-term outcomes and cost-effectiveness considerations remain unexplored in the current literature.

## 5 Conclusion

In conclusion, this meta-analysis demonstrates that SJOV significantly enhances the safety of bronchoscopic procedures by markedly reducing the incidence of hypoxemia and the need for rescue interventions, while concurrently improving intraoperative oxygenation. The technique presents a favorable safety profile, with the most common adverse effects being transient mucosal symptoms that are outweighed by its clinical benefits. By effectively addressing the physiological challenges of the shared airway, SJOV emerges as a particularly valuable strategy for high-risk patients undergoing bronchoscopy. However, given the unclear reporting of allocation concealment and blinding across included trials, these findings should be interpreted with appropriate caution. Future studies should aim to standardize optimal device parameters, validate these findings in broader populations, and evaluate long-term outcomes and cost-effectiveness to guide its widespread clinical adoption.

## Data Availability

The original contributions presented in the study are included in the article/supplementary material, further inquiries can be directed to the corresponding author.
